# The Dangers of Big Panels

**Published:** 1919-08-23

**Authors:** 


### THE DANGERS OF BIG PANELS.

It is a recognised truth that no person can give of his best unless he devotes his undivided attention to the work in hand, and in so vital a matter as the health of the community the doctor should be untrammelled in his efforts to cure suffering humanity. During the war the scarcity of medical men compelled those left at home to perform double, or even treble, their pre-war amount of work, and many of them underwent severe physical and mental strain. But with the return of doctors from the theatres of war, there cannot be the same necessity for individual press of work. When the panel system was first mooted a great number of medical men found themselves bitterly opposed to its principles, because they felt that there was a great danger of a spirit of commercialism gaining a hold on the minds of those who took panel patients. Most of us will remember the meetings of protest which were held in different parts of the country, and it was generally considered that the benefits accruing to those patients on the panels would become less in inverse ratio to the size of any one panel.

All medical men in general practice of some years' standing know that a great number of their patients come to consult them when they have little or nothing the matter with them, and especially is this the case under the panel system. The diagnosis and treatment of such cases are often regarded as comparatively easy matters, and the time spent on examination may be, and often is, extremely short; but here lies a grave source of danger, for even the cleverest and most experienced man may be led into committing a serious mistake through making a hurried diagnosis following on such a brief inspection. There are many cases which, under a more or less cursory examination, are often classified as of the so-called functional or hysterical types, whereas under a more detailed and painstaking examination some organic lesion may ultimately be found. To detect such cases as these requires all the undivided skill and attention of the diagnostician, who cannot possibly arrive at a satisfactory conclusion in any hurried manner.

Every thinking man knows his own limitations, and to act conscientiously towards his patients he must, as far as he possibly can, keep himself within those limitations. His one aim and object must be to try and find a sure preventive against, and cure for, the diseases which he is called upon to treat. Many and serious mistakes have been made, both in hospital and private practice, by adopting the principle of hurry; it is very regrettable, and almost criminal, that any medical man should allow the thought of the financial position of his patient to influence in any degree the amount of trouble and time which he expends over that case.

But, like everyone else, the doctor must live. He rarely can afford to be a pure philanthropist, and he is too often compelled by force of circumstances to accept more work than he can conscientiously carry out. To this class must be added a minority who throw aside their principles of higher ethics, and enter the profession with the avowed object of carrying on their practices on the lines of an ordinary trading concern, thereby lowering this great profession in the eyes of the world.

We agree with the *Times* that the number of persons on any one panel should not exceed a certain fixed amount—we should put the figure at 2,000—and that for his work in connection with this panel the doctor should receive a stipend sufficiently large to enable him to attend to his duties untrammelled by financial worries, and thus act in the best interests of his patients.

